# Evaluation of the expression levels of *BRAF*^*V600E*^ mRNA in primary tumors of thyroid cancer using an ultrasensitive mutation assay

**DOI:** 10.1186/s12885-020-06862-w

**Published:** 2020-05-01

**Authors:** Tien Viet Tran, Kien Xuan Dang, Quynh Huong Pham, Ung Dinh Nguyen, Nhung Thi Trang Trinh, Luong Van Hoang, Son Anh Ho, Ba Van Nguyen, Duc Trong Nguyen, Dung Tuan Trinh, Dung Ngoc Tran, Arto Orpana, Ulf-Håkan Stenman, Jakob Stenman, Tho Huu Ho

**Affiliations:** 1 103 Military Hospital, Vietnam Military Medical University, Hanoi, Vietnam; 2 Minerva Foundation Institute for Medical Research, Helsinki, Finland; 3 Department of Genomics and Cytogenetics, Institute of Biomedicine and Pharmacy (IBP), Vietnam Military Medical University, 222 Phung Hung street, Ha Dong district, Hanoi, Vietnam; 4 Institute of Biomedicine and Pharmacy (IBP), Vietnam Military Medical University, Hanoi, Vietnam; 5 Oncology Centre, 103 Military Hospital, Vietnam Military Medical University, Hanoi, Vietnam; 6 School of Medicine and Pharmacy, Vietnam National University, Hanoi, Vietnam; 7 Pathology Department, 108 Military Central Hospital, Hanoi, Vietnam; 8 Department of Pathology, 103 Military Hospital, Vietnam Military Medical University, Hanoi, Vietnam; 9 Laboratory of Genetics, HUSLAB, Helsinki University Central Hospital, Helsinki, Finland; 10 Department of Clinical Chemistry, Medicum, Helsinki University Hospital, University of Helsinki, Helsinki, Finland; 11 Department of Women’s and Children’s Health, Karolinska Institutet, Stockholm, Sweden; 12 Department of Medical Microbiology, 103 Military Hospital, Vietnam Medical University, Hanoi, Vietnam

**Keywords:** Thyroid cancer, BRAF mutation, mRNA mutation assay, Diagnosis

## Abstract

**Background:**

The *BRAF*^*V600E*^ gene encodes for the mutant BRAF^V600E^ protein, which triggers downstream oncogenic signaling in thyroid cancer. Since most currently available methods have focused on detecting *BRAF*^*V600E*^ mutations in tumor DNA, there is limited information about the level of *BRAF*^*V600E*^ mRNA in primary tumors of thyroid cancer, and the diagnostic relevance of these RNA mutations is not known.

**Methods:**

Sixty-two patients with thyroid cancer and non-malignant thyroid disease were included in the study. Armed with an ultrasensitive technique for mRNA-based mutation analysis based on a two step RT-qPCR method, we analysed the expression levels of the mutated *BRAF*^*V600E*^ mRNA in formalin-fixed paraffin-embedded samples of thyroid tissues. Sanger sequencing for detection of *BRAF*^*V600E*^ DNA was performed in parallel for comparison and normalization of *BRAF*^*V600E*^ mRNA expression levels.

**Results:**

The mRNA-based mutation detection assay enables detection of the *BRAF*^*V600E*^ mRNA transcripts in a 10,000-fold excess of wildtype *BRAF* counterparts. While *BRAF*^*V600E*^ mutations could be detected by Sanger sequencing in 13 out of 32 malignant thyroid cancer FFPE tissue samples, the mRNA-based assay detected mutations in additionally 5 cases, improving the detection rate from 40.6 to 56.3%. Furthermore, we observed a surprisingly large, 3-log variability, in the expression level of the *BRAF*^*V600E*^ mRNA in FFPE samples of thyroid cancer tissue.

**Conclusions:**

The expression levels of *BRAF*^*V600E*^ mRNA was characterized in the primary tumors of thyroid cancer using an ultrasensitive mRNA-based mutation assay. Our data inspires further studies on the prognostic and diagnostic relevance of the *BRAF*^*V600E*^ mRNA levels as a molecular biomarker for the diagnosis and monitoring of various genetic and malignant diseases.

## Background

Thyroid cancer is the most frequent endocrine cancer and the fourth most common cancer in women, with a worldwide annual incidence of 3.1% [1]. One of the most important events in the progression of thyroid cancer is the occurrence of the *BRAF*^*V600E*^ mutation, which can be detected in 29–83% of cases [2]. This somatic missense mutation at the nucleotide position 1799 T > A results in substitution of glutamic acid (E) for valine (V) at codon 600 [3]. The constitutively active BRAF^V600E^ protein transduces mitogenic signals from the cell membrane to the nucleus, thus leading the deregulation of cell proliferation and oncogenesis [4–6]. Detection of the *BRAF*^*V600E*^ mutation in DNA has been consistently reported as a useful prognostic and diagnostic biomarker in thyroid cancer [7, 8].

Up to date, there are several methods for *BRAF*^*V600E*^ DNA mutation testing, including Sanger sequencing [9], pyrosequencing [10], allele-specific PCR (AS-PCR) [11], high resolution melting (HRM) analysis [12], and COLD-PCR [13]. These methods vary in sensitivity, specificity, assay complexity and costs. Although Sanger sequencing exhibits highly reliable and specific outputs, it suffers from the risk of handling contamination, costly, time consuming, and a relatively low sensitivity, requiring a 7–20% mutant allele frequency for reliable detection [9]. In comparison, allele-specific PCR (AS-PCR), high resolution melting analysis, COLD-PCR have been reported to have an analytical sensitivity ranging from 0.1 to 2%, 1 and 3.1%, respectively [11–13].

As an alternative to DNA-based mutation assays, antibody-based test using the monoclonal antibody VE1 has recently been reported to specifically detect the presence of mutant BRAF^V600E^ protein in tumor specimens [14]. This IHC detection enables visualization of the distribution of BRAF^V600E^ mutant protein at a single-cell level with semiquantitative readout of protein abundance, thus improving sensitivity and specificity in comparison to DNA-based tests. High heterogeneity of BRAF^V600E^ expression, causing false negatives, and restrictions for other BRAF variants are the main weaknesses of this method [15].

Despite various methods for *BRAF*^*V600E*^ mutation analysis at both the DNA and protein levels, there is still limited information regarding the mRNA level of the mutated *BRAF*^*V600E*^ allele in primary thyroid cancer tumors. The use of mRNA as a template allows for measuring mRNA levels of the mutated and wildtype genes, which, like protein-based testing, might reflect the functional consequences of the mutated genes in cell and tissue more accurately than assays based on detection of the mutation in DNA only. Furthermore, the number of mRNA molecules of a moderately or highly expressed gene, often exceeds the copy number of DNA counterparts by several orders of magnitude, which allows an increased sensitivity of detection.

In this study, we performed *BRAF*^*V600E*^ mutation analysis using formalin-fixed paraffin-embedded (FFPE) samples of thyroid tissues from 62 patients, using an mRNA-based mutation assay with improved sensitivity to clarify the diagnostic and prognostic relevance of the level of mutant *BRAF*^*V600E*^ in relation to wildtype *BRAF* alleles at the mRNA level.

## Methods

### Patient samples and nucleic acid extraction

FFPE tissue samples from 62 patients were obtained from the Department of Pathology, 103 Military Hospital, Hanoi, Vietnam (Table S2). Multiple 10 μm-thickness sections that contain 10 mg of FFPE tissue were collected, then deparaffinized by mineral oil before extraction of nucleic acids. RNA was extracted using GenElute™ FFPE RNA Purification Kit (Sigma – Aldrich, Canada), and DNA was extracted using QIAamp DNA FFPE Tissue Kit (Qiagen, Germany), according to the manufacturers’ instructions. The nucleic acid concentration was determined using an ND-1000 spectrophotometer (NanoDrop, Walmington, DE). In-vitro transcribed mRNA of the mutated *BRAF*^*V600E*^ variant (mutant mRNA) and wildtype *BRAF* (wildtype mRNA) was utilized for determination of the sensitivity of *BRAF*^*V600E*^ mRNA-based mutation assay [16].

### Overview of the mRNA-based mutation assay

**Fig. 1:**
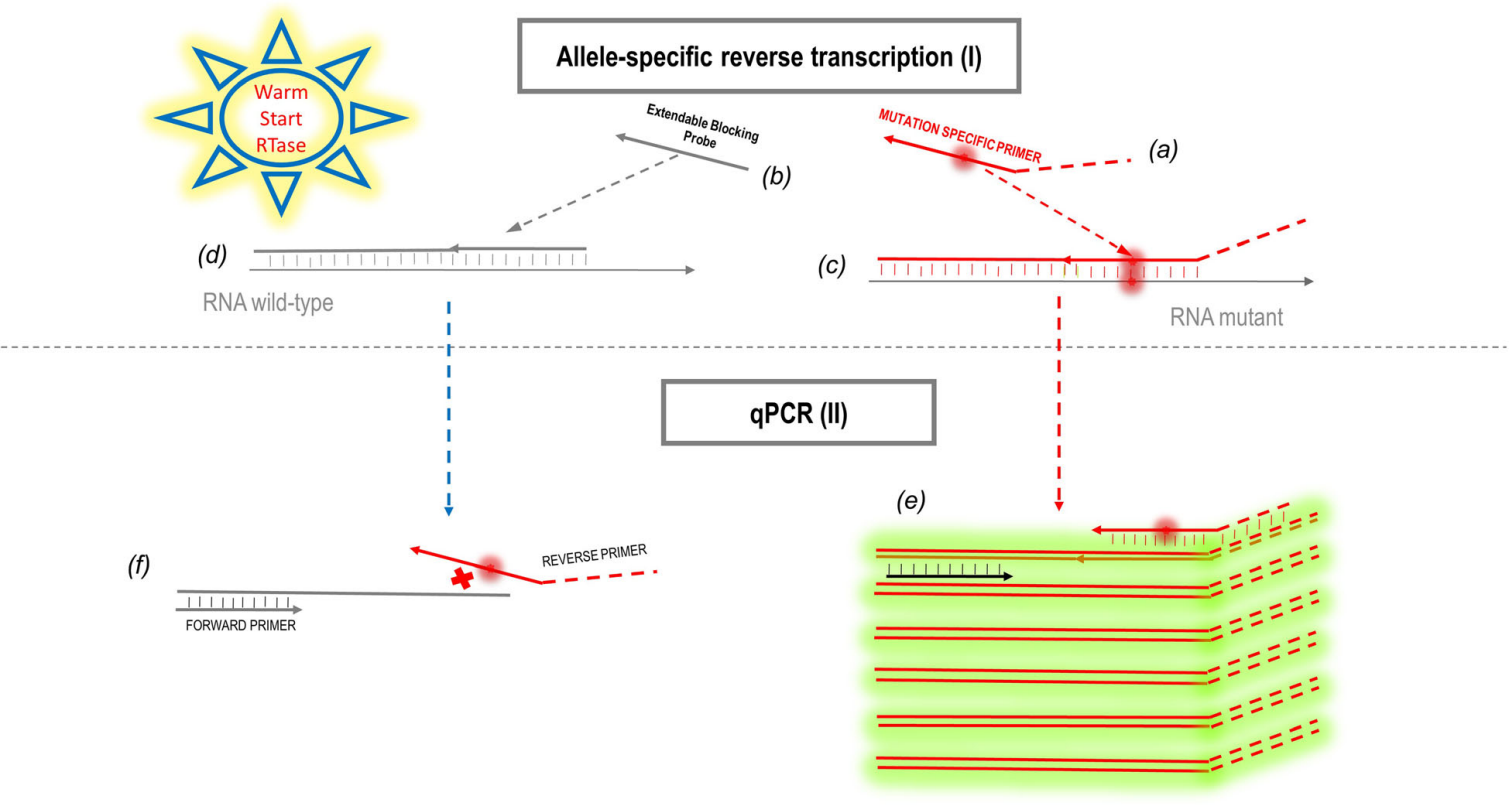
Overview of the *BRAF*^*V600E*^ mRNA mutation detection assay. Mutant *BRAF*^*V600E*^ mRNA was detected in a two-step qPCR reaction as follows: I) A mutation-specific reverse transcription, utilizing a warmstart reverse transcriptase that is activated at relatively high temperature (40^o^-50 °C), in combination with an extendable wildtype-blocking probe and a 5′-tailed *BRAF*^*V600E*^ mutation-specific primer; II) selective qPCR amplification of cDNA derived from mutant *BRAF*^*V600E*^ mRNA

The principle of Extendable Blocking Probe-Reverse Transcription (ExBP-RT) assay, which was recently developed in our laboratory [16], utilizes an extendable wildtype-blocking probe that competes with a mutation-specific primer for annealing and extension of the mutant and corresponding wildtype mRNA during reverse transcription (Fig. 1). This allows for mutation-specific reverse transcription and subsequent selective qPCR amplification of cDNA derived from mutated mRNA. Improvements to the original protocol include optimal design of the mutation-specific primer and a recently developed warmstart reverse transcriptase enzyme which is activated above 40 °C (Table S2). A slow cooling toward the optimal annealing temperature during reverse transcription ensures that correct priming at a higher temperature occurs temporally prior to any possible mispriming event (Fig. 1c, d). The mutated BRAF^*V600E*^ mRNA template can thus, be selectively amplified in a highly specific RT-qPCR assay (Fig. 1e).

### Primer and probe design for the *BRAF*^*V600E*^ mRNA-based mutation assay

In order to segregate mutant and wildtype mRNA transcripts during reverse transcription, we designed a mutation-specific primer (Fig. 1a) and an extendable wildtype-blocking probe (Fig. 1)b with a sequence of 12–14 nucleotides, complementary to the mutant and corresponding wildtype mRNA at the mutation site (5′- AGATTTC**A**CTGTAG-3′). A 5′-tail consisting of 10 nucleotide sequence, unrelated to the target gene, was incorporated in the mutation-specific primer (5′-CTCTCCCGTTGATTTC**T**CTGTA-3′). The mutation-specific primer was also used as the reverse primer during qPCR, allowing for selective amplification of cDNA derived from mutant mRNA.

### Two step RT-qPCR for detection of expressed *BRAF*^*V600E*^ mutation

Reverse transcription was carried out in a 10 μl reaction containing 1X buffer, 1.875 U reverse transcriptase (WarmStart_®_ Reverse Transcriptase, NEB, USA), 0.5 mM of each dNTP, 0.125 μM mutation-specific primer, 0.8 μM extendable wildtype-blocking probe, and mRNA template. The cDNA synthesis was performed at 50 °C for 5 min, after which, the temperature was gradually decreased to 40 °C, 1 °C per minute with a final enzyme inactivation step at 80 °C for 15 min. Following reverse transcription, 2 μl of cDNA was transferred to the qPCR reaction. qPCR was performed in duplicate using the Rotor Gene Q realtime detection system (Qiagen, Germany) in a 20 μl reaction containing 1x QuantiTect SYBR Green master mix (Qiagen), 0.8 μM forward primer (5′- CATGAAGACCTCACAGTAAA-3′), reverse primer (5′-CTCTCCCGTTGATTTC**T**CTGTA-3′), and 2 μl cDNA template. The cycling protocol included denaturation at 95 °C for 15 min, followed by 45 cycles of 94 °C for 15 s, 63 °C for 30 s and 72 °C for 30 s. A parallel wildtype *BRAF* SYBR qPCR was performed in duplicate to control for mRNA extraction, as well as for measurement of the wildtype *BRAF* mRNA level (forward primer: 5′- CATGAAGACCTCACAGTAAA-3′; and the reverse primer: 5′- GATTTCACTGTAGCTAGACC-3′).

### Determination of the sensitivity for detection of *BRAF*^*V600E*^ mRNA mutation

The sensitivity of the mRNA-based mutation assay for detecting mutant mRNA transcripts in a background of corresponding wildtype transcripts was determined by comparing the amount of PCR product formed in a first reaction containing 10^7^ copies of in-vitro transcribed wildtype *BRAF* mRNA as a template, with the amount of PCR product created in a second reaction containing the same amount of transcribed mutant *BRAF*^*V600E*^*m*RNA. The threshold cycle value (Ct value) was identified automatically during qPCR amplification by the Rotor Gene Q system (Qiagen, Germany). The ratio of products formed in the first reaction and second reaction were determined by quantitative PCR based on the difference in Ct values derived from the two reactions (∆Ct_wt-mt_ = Ct_wildtype_ − Ct_mutant_). The sensitivity of the mRNA-based mutation assay for *BRAF*^*V600E*^ mutation, expressed as percentage, was calculated as 2^-∆Ct^ × 100%, which corresponds to the lowest fraction of mutant transcripts to be detected as a distinct signal in a background signal derived from cross-priming of the wildtype template.

### DNA sequencing

DNA extracted from clinical FFPE samples were amplified by PCR in 20 μl reactions of Kapa HiFi HotStart ReadyMix (Kapa Biosystems, USA) containing 1X buffer, 0.5 μM forward primer (5′-CATGAAGACCTCACAGTAAA-3′), 0.5 μM reverse primers (5′- ACTGTTCAAACTGATGGGACCCAC − 3′), and DNA template. PCR was performed by denaturation at 95 °C for 5 min, followed by 40 cycles of 98 °C for 30 s, 60 °C for 30 s, 72 °C for 30 s with a final extension at 72 °C for 1 min, using a conventional PCR thermal cycler Eppendorf vapo.protect (Eppendorf, Germany). PCR products were purified by ExoSAP-IT® PCR Product Cleanup (Affimetrix, USA) and subsequently subjected to Sanger sequencing using ABI 3130xl Genetic Analyzer system (Applied Biosystem, USA) with the reverse primer as sequencing primer.

### Statistical analysis

Cohen’s Kappa coefficient and McNemar’s chi-square tests were used to compare the performance of two tests, mRNA-based mutation assay and Sanger sequencing method.

## Results

### Patient samples

Sixty-two patients were included in the study. Thirty-two of these had been diagnosed with thyroid cancer and 30 patients with benign thyroid disease. Out of the 32 thyroid carcinoma samples, 24 (75%) were papillary thyroid cancer (Table 1 and Table S1). Ethics approval and consent to participate in the study was obtained in accordance with the Declaration of Helsinki.

**Table 1:** Clinicopathologic parameters in patients with thyroid diseases

Clinicopathologic parameters		Frequencies	
		Number	Percentage (%)
Sex	Male	7	11.3
	Female	55	88.7
Histology of malignant tumours	Papillary	24	75.0
	Follicular	6	18.8
	Mixed Papillary – Follicular variant	1	3.1
	Thyroid Adenocarcinoma	1	3.1
Histology of benign tumours	Nontoxic single thyroid nodule	9	30.0
	Benign neoplasm of thyroid gland	20	66.7
	Basedow with euthyroid phase stage	1	3.3

### Sensitivity of the *BRAF*^*V600E*^ mRNA mutation detection assay

**Fig. 2:**
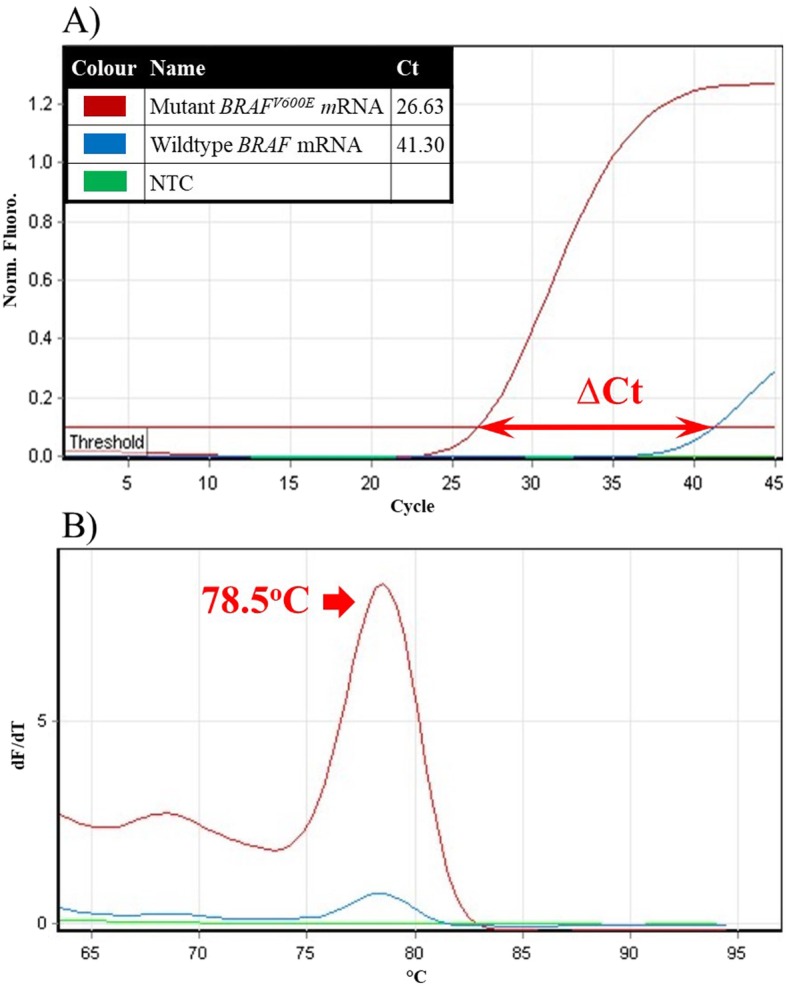
Detection sensitivity for *BRAF*^*V600E*^ mutation in mRNA. The sensitivity of a novel mRNA based mutation assay for *BRAF*^*V600E*^ was determined using 10^7^ copies of in vitro transcribed mRNA containing the *BRAF*^*V600E*^ mutation and the same amount of corresponding wildtype mRNA as templates: **a** Amplification signal from mutant *BRAF*^*V600E*^ mRNA (red line), wildtype *BRAF* mRNA (blue line) and no-template control-NTC (green line); **b**) Corresponding melting peaks of the amplification products

The sensitivity of mRNA-based mutation assay was determined using in vitro transcribed mutant *BRAF*^*V600E*^ and corresponding wildtype *BRAF* mRNA as templates (Fig. 2). The amplification product derived from qRT-PCR amplification of 10^7^ copies of the mutant *BRAF*^*V600E*^ mRNA was detected 14.67 cycles earlier than the amplification product derived from wildtype *BRAF* mRNA. The signal generated from the amplification of wildtype *BRAF* mRNA represents the cross-priming of mutation-specific primer to the wildtype *BRAF m*RNA template. The difference in threshold values, delta Ct, thus corresponds to a cross-priming efficiency of approximately 0.005% of the specific priming efficiency (2^-∆Ct^ × 100% = 2^–14.67^ × 100%). As a result, the mRNA-based mutation assay can detect the *BRAF*^*V600E*^ mutation in mRNA with frequency of 0.01%, or in other words, in the presence of a 10,000-fold excess of the wildtype *BRAF* counterpart.

### Detection of the *BRAF*^*V600E*^ mutation in mRNA and DNA from benign and malignant thyroid FFPE tissue samples

**Fig. 3:**
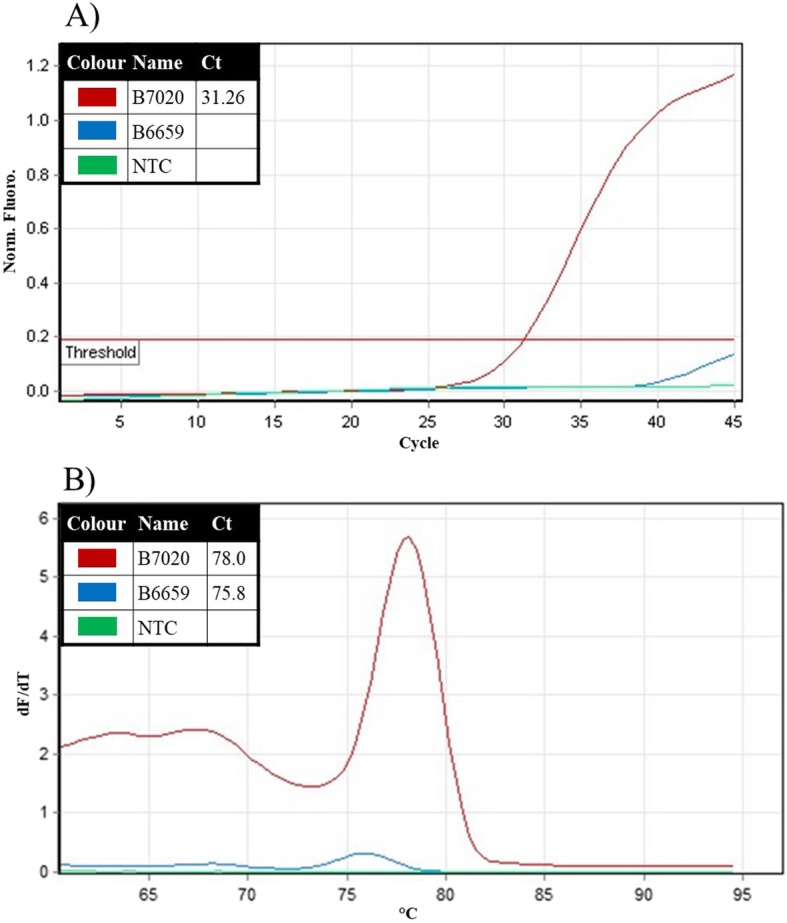
Detection of *BRAF*^*V600E*^ mutation in mRNA from clinical FFPE samples. *BRAF*^*V600E*^ mRNA based mutation assay was utilized for ultrasensitive detection of the *BRAF*^*V600E*^ mutation in mRNA isolated from clinical FFPE specimens of thyroid cancer and non-malignant thyroid disease. **a** Amplification signals from a sample containing mutant *BRAF*^*V600E*^ mRNA (B7020 - red line), a sample without mutant *BRAF*^*V600E*^ mRNA (B6659 - blue line) and no-template control (NTC - green line); **b** Corresponding melting peaks of the amplification products

**Fig. 4:**
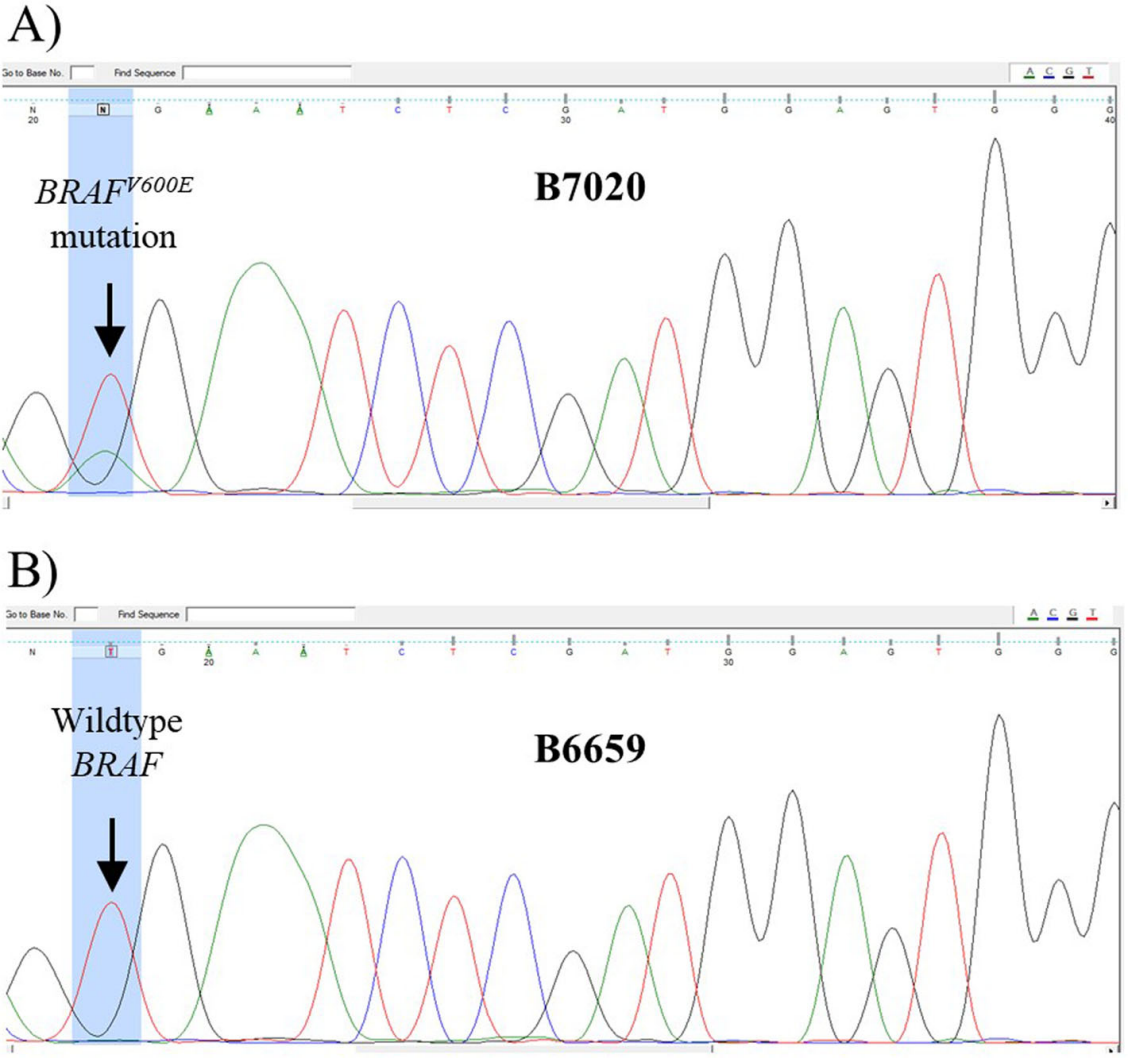
Detection of the *BRAF*^*V600E*^ mutation in FFPE samples using DNA sequencing. Sanger DNA sequencing was used as a reference method to detect the *BRAF*^*V600E*^ mutation in clinical FFPE specimens from patients with thyroid cancer and non-malignant thyroid disease. **a** Sequencing chromatogram showing two peaks (red and green) at the nucleotide position of interest for a sample with the *BRAF*^*V600E*^ mutation (B7020), and **b** single peak (red) for a sample with wild type BRAF only (B6659)

The clinical applicability of the mRNA-based mutation assay for *BRAF*^*V600E*^ mRNA was evaluated by analyzing nucleic acids isolated from FFPE tissue samples of thyroid tumors and non-malignant thyroid disease, and comparing results with direct sequencing (Fig. 3). *BRAF*^*V600E*^ mRNA was detected in 18 out of 32 thyroid cancer samples (56.3%) with the *BRAF*^*V600E*^ mRNA based mutation assay. In comparison, *BRAF*^*V600E*^ DNA was detected by Sanger sequencing in only 13 (40.6%) of these 18 samples (Fig. 4). The presence of *BRAF*^*V600E*^ mRNA could be confirmed in all 13 FFPE samples in which the mutation was detected by in DNA, by Sanger sequencing. The Cohen’s Kappa coefficient of 0.695 reveals the substantial agreement between the current mRNA-based mutation assay and Sanger sequencing method, in detecting the *BRAF*^*V600E*^ mutation in thyroid cancer tissue samples. On the other hand, the McNemar’s chi-square test shows a two-tailed *P* value of 0.0736, suggesting a borderline significant difference between two tests in the detection of the *BRAF*^*V600E*^ mutation. No *BRAF*^*V600E*^ mutation was detected either in mRNA by the *BRAF*^*V600E*^ mRNA-based mutation assay, or in DNA by Sanger sequencing, in any of the 30 FFPE samples of benign thyroid tissues, indicating a high specificity of both assays.

### Determination of relative expression levels of the *BRAF*^*V600E*^ mRNA versus wildtype *BRAF* mRNA

We further investigated the allele-specific expression of the mutant and wildtype alleles of the *BRAF* gene in the 13 thyroid cancer tissue samples with *BRAF*^*V600E*^ mutation detected in both DNA and mRNA (Table S1). The relative abundance of mutant versus wildtype alleles at the DNA levels was estimated using the peak heights (H) at the nucleotide position of interest (1799 T > A) on a direct sequencing chromatogram: R^*DNA*^ = H^*BRAFV600E*^ / H^*BRAFwildtype*^. Similarly, the relative abundance of mutant versus wildtype alleles at the mRNA levels was estimated using the delta Ct value (ΔCt) between the mutant and wildtype signals in mRNA-based mutation assays: R^*RNA*^ = 1/2^ΔCt(*BRAFV600E-BRAFwildtype)*^. The relative abundance of the mutated *BRAF*^*V600E*^ allele in DNA was relatively constant, in the range 0.170–0.703. On the mRNA levels, however, the relative abundance of the mutated *BRAF*^*V600E*^ alleles varied in the range of 0.001–0.429. The observed log (R^*RNA*^/R^*DNA*^) ratio was in the range − 2.48 - 0.35, corresponding to almost 3 log differences in expression levels of the mutated *BRAF*^*V600E*^ alleles versus the wildtype *BRAF* counterparts in these tissue samples.

## Discussion

In spite of functional genomics being an appealing approach for studying the relationship between genes and diseases, there is currently no data available regarding the specific mRNA expression of the *BRAF*^*V600E*^ mutation in different cancer tissues. Many papillary thyroid cancers possess a mutated *BRAF* gene, most commonly the point mutation T1799A or *BRAF*^*V600E*^, which activates the MAPK pathway causing a loss of control of cellular proliferation, triggering the oncogenesis of thyroid gland [6, 17, 18]. We detected *BRAF*^*V600E*^ mutations on the mRNA level in 56,3% (18/32) and on the DNA level in 40,6% (13/32) of thyroid cancer patients, which is roughly in concordance with the prevalence reported by a number of studies [2, 19–22]. The mRNA-based mutation detection assay, thus contributed to a 28% improvement in the sensitivity of detection, whereas the specificity of both the mRNA- and DNA-based assays was 100%. According to a number of studies, the prognostic relevance of *BRAF*^*V600E*^ mutation still remains controversial in papillary thyroid carcinoma [23–26]. While the *BRAF*^*V600E*^ mutation is not an independent predictor of poor outcome, the presence of the mutation is valuable for determining whether certain high-risk patients, in a relapse or primary metastatic setting, could be eligible for targeted *BRAF* inhibitor therapy with any of the currently available drugs, such as lenvatinib, vemurafenib or sorafenib [27]. Also, the presence of the *BRAF*^*V600E*^ mutation in the primary tumor tissue opens possibilities for monitoring of the disease using liquid biopsy techniques.

Sanger sequencing is currently considered as the gold standard for point mutation detection, primarily due to the possibility to analyze a multitude of different mutations simultaneously. Drawbacks of this method are a relatively long, 2–3 day turn-around time as well as a relatively low sensitivity, limiting the detection of mutated alleles below a frequency of 7–20% [9]. Subsequently, a significant number of low-level mutations will remain undetected primarily due to tumor tissue heterogeneity and a relatively low frequency of mutated alleles. In our study, Sanger sequencing failed to detect the *BRAF*^*V600E*^ mutation in 5 out of 18 samples, which were positive with *BRAF*^*V600E*^ mRNA. *BRAF*^*V600E*^ mRNA should, by definition, only be detected in a subgroup of patients haboring *BRAF*^*V600E*^ mutation in DNA. In spite of this, the novel mRNA-based assay detected *BRAF*^*V600E*^ mutations at a higher frequency than Sanger sequencing in FFPE samples from the same cohort of thyroid cancer patients. We speculate that this discrepancy might partially be explained by the superior technical sensitivity of the mRNA-based assay compared to direct sequencing, but also by the higher copy number of *BRAF*^*V600E*^ mRNA transcripts in comparison to that of *BRAF*^*V600E*^ DNA in thyroid cancer cells.

We also analyzed the relative level of the mutant *BRAF*^*V600E*^ allele in the thyroid cancer FFPE tissue samples separately on the DNA and mRNA expression level. On the DNA level the relative abundance of *BRAF*^*V600E*^ versus wildtype *BRAF* ranged between 0.170–0.703, while the variation in the relative abundance of the respective alleles was much wider on the mRNA level, in the range of about 3 logs (0.001–0.429). This suggests that the expression level of the *BRAF*^*V600E*^ gene can be highly variable in thyroid cancer and maybe in other cancers as well. The level of *BRAF*^*V600E*^ mRNA expression can to some extent be predictive of the subsequent expression of a mutant protein, and this may provide some insights to the role of BRAF mutations in cancer progression and prognosis. Nevertheless, the number of mRNA copies does not always reflect the functional protein expression level due to several post-transcriptional factors. A challenge for gene expression studies on mutation-dependent diseases is to innovate and implement integrative methodologies to analyze mRNA/protein expression in parallel.

Mutation detection at the mRNA level benefits from a higher copy number of mutated mRNA transcripts per cancer cell compared to the number of mutated DNA copies. Detection of the *BRAF*^*V600E*^ mutations in mRNA without prior amplification has been demonstrated using a nanomechanical sensor comprising of microcantilever arrays coated with titanium and gold in combination with with a probe oligonucleotide and non-specific reference oligonucleotides [28]. This ultrasensitive device enables detection of mRNA at a concentration of 20 ng/μl and recognition of mutated *BRAF* DNA in a 50-fold excess of the wildtype background. In addition, there have been several improvements to previously existing amplification technologies, most recently by using artificial mismatched nucleotides on allele-specific primers to improve segregation between the respective alleles and externally added controller sequences [29]. Many other sensitive mutation detection assays based on the principle of allele-specific PCR have been described [30–32]. All of these technologies are, however, hampered by cross priming during amplification, leading to a decay in the discriminating power during the amplification process [33, 34]. The rate of cross-priming is dependent on the nucleotide used for discrimination between the alleles. In particular, PCR product yields have been shown to decrease by 20-fold for A:A mismatches, whereas mismatches involving T have minimal effect on PCR product yield [35]. Therefore, the design of AS-PCR assays for detection of the *BRAF*^*V600E*^ (1799 T > A) mutation, which involves A:A or T:T mismatches, is inherently challenging, restricting assay sensitivity to about 0.1% at best [12, 13, 21, 36–39]. In contrast, the ExBP-RT technique used in this study discriminates between wild type and mutant alleles during a single cycle of reverse transcription, completely eliminating the problem of decay of sensitivity during subsequent qPCR amplification [16].

## Conclusions

In conclusion, we have successfully established a novel assay for ultrasensitive detection and quantification of the *BRAF*^*V600E*^ mRNA in FFPE tissue from thyroid cancer. This assay not only reveals the presence of the *BRAF*^*V600E*^ mutation, but also the level of the mutated *BRAF*^*V600E*^ mRNA. This approach opens new possibilities to study the functional consequences of mRNA expression of mutated genes and the potential clinical utility of mutation detection in mRNA, as a novel biomarker in various types of cancer and genetic diseases.

## Supplementary information

**Additional file 1: Table S1.** Clinicopathologic and molecular data of novel mRNA-based assay and Sanger sequencing for *BRAF*^*V600E*^ expression of thyroid cancer cases. 

**Additional file 2: Table S2.** Improvements of current mRNA-based mutation assay in comparison to the original assay of Extendable blocking probe - reverse transcription (ExBP-RT).

## Data Availability

The datasets used and/or analysed during the current study available from the corresponding author on reasonable request.

## Abbreviations

BRAF: V-raf murine sacoma viral oncogene homolog B
ExBP-RT: Extendable blocking probe –reverse transcription
FFPE samples: Formalin-fixed paraffin-embeded samples
IHC: Immunohistochemistry
MAPK: Mitogen-activated protein kinase
