# Supplementary material for: Evaluation of the expression levels of BRAFV600E mRNA in primary tumors of thyroid cancer using an ultrasensitive mutation assay
Source: BMC Cancer. 2020 May 1;20:368. doi: 10.1186/s12885-020-06862-w (PMC7195771; doi:10.1186/s12885-020-06862-w)
Supplement: Supplementary file 1 — Additional file 1: Table S1. Clinicopathologic and molecular data of novel mRNA-based assay and Sanger sequencing for BRAFV600E expression of thyroid cancer cases. [file 12885_2020_6862_MOESM1_ESM.pdf]

Table S1. Clinicopathologic and molecular data of novel mRNA-based assay and Sanger sequencing for *BRAF*^{V600E} expression of thyroid cancer cases.

Case No.	Sex	Tumor type	Molecular data				
			Novel mRNA-based assay[b]		Sequencing[c]		Log (R^{RNA}/R^{DNA})
			ΔCt (mt-wt)	R^{RNA}	nt 1799	R^{DNA}	
1	1	Malignant	2.58	0.16724	1799T>A	0.703	-0.623
2	1	Malignant	5.71	0.0191	1799T>A	0.6	-1.497
3	1	Malignant	4.97	0.03191	1799T>A	0.48	-1.177
4	1	Malignant	1.22	0.42928	1799T>A	0.378	0.056
5	1	Malignant	1.81	0.28519	1799T>A	0.368	-0.11
6	1	Malignant	2.84	0.13966	1799T>A	0.313	-0.35
7	2	Malignant	10	0.00098	1799T>A	0.295	-2.48
8	1	Malignant	2.15	0.22531	1799T>A	0.28	-0.094
9	1	Malignant	4.87	0.0342	1799T>A	0.265	-0.889
10	1	Malignant	4.35	0.04904	1799T>A	0.228	-0.666
11	1	Malignant	1.41	0.37631	1799T>A	0.208	0.259
12	1	Malignant	6.56	0.0106	1799T>A	0.178	-1.224
13	1	Malignant	3	0.125	1799T>A	0.17	-0.134
14	2	Malignant	2.38	0.19211	1799T	0	n.a.
15	1	Malignant	4.08	0.05913	1799T	0	n.a.
16	1	Malignant	6.52	0.0109	1799T	0	n.a.
17	1	Malignant	7.62	0.00508	1799T	0	n.a.
18	1	Malignant	8.15	0.00352	1799T	0	n.a.
19	1	Malignant	wt	0	1799T	0	n.a.
20	1	Malignant	wt	0	1799T	0	n.a.
21	1	Malignant	wt	0	1799T	0	n.a.
22	1	Malignant	wt	0	1799T	0	n.a.
23	1	Malignant	wt	0	1799T	0	n.a.
24	1	Malignant	wt	0	1799T	0	n.a.
25	1	Malignant	wt	0	1799T	0	n.a.
26	1	Malignant	wt	0	1799T	0	n.a.
27	1	Malignant	wt	0	1799T	0	n.a.
28	1	Malignant	wt	0	1799T	0	n.a.
29	1	Malignant	wt	0	1799T	0	n.a.
30	1	Malignant	wt	0	1799T	0	n.a.
31	1	Malignant	wt	0	1799T	0	n.a.
32	1	Malignant	wt	0	1799T	0	n.a.
33	1	Benign	wt	0	1799T	0	n.a.
34	1	Benign	wt	0	1799T	0	n.a.
35	2	Benign	wt	0	1799T	0	n.a.
36	1	Benign	wt	0	1799T	0	n.a.
37	1	Benign	wt	0	1799T	0	n.a.
38	2	Benign	wt	0	1799T	0	n.a.
39	1	Benign	wt	0	1799T	0	n.a.
40	1	Benign	wt	0	1799T	0	n.a.

41	1	Benign	wt	0	1799T	0	n.a.
42	1	Benign	wt	0	1799T	0	n.a.
43	2	Benign	wt	0	1799T	0	n.a.
44	2	Benign	wt	0	1799T	0	n.a.
45	1	Benign	wt	0	1799T	0	n.a.
46	1	Benign	wt	0	1799T	0	n.a.
47	1	Benign	wt	0	1799T	0	n.a.
48	1	Benign	wt	0	1799T	0	n.a.
49	1	Benign	wt	0	1799T	0	n.a.
50	1	Benign	wt	0	1799T	0	n.a.
51	1	Benign	wt	0	1799T	0	n.a.
52	1	Benign	wt	0	1799T	0	n.a.
53	1	Benign	wt	0	1799T	0	n.a.
54	1	Benign	wt	0	1799T	0	n.a.
55	1	Benign	wt	0	1799T	0	n.a.
56	1	Benign	wt	0	1799T	0	n.a.
57	1	Benign	wt	0	1799T	0	n.a.
58	1	Benign	wt	0	1799T	0	n.a.
59	1	Benign	wt	0	1799T	0	n.a.
60	2	Benign	wt	0	1799T	0	n.a.
61	1	Benign	wt	0	1799T	0	n.a.
62	1	Benign	wt	0	1799T	0	n.a.

[a]All cases were diagnosed with differentiated thyroid cancer.
[b]Novel mRNA-based assay was used to calculate the relative abundance of mutant versus wildtype allele at the mRNA level using the delta Ct value (ΔCt) between the mutant and wildtype signals: $R^{RNA} = 1/2\Delta Ct(\textit{BRAF}^{V600E}\text{-}\textit{BRAF}\text{ wildtype})$.
[c]Sanger sequencing was used to calculate the relative abundance of mutant versus wildtype allele at the DNA level using the peak heights (H) at the nucleotide position of interest (1799T>A) on a DS chromatogam: $R^{DNA} = H^{BRAFV600E} / H^{BRAFwildtype}$.
wt: wild-type; mt: mutant; n.a: no applicable.
