# Supplementary material for: Evaluation of the expression levels of BRAFV600E mRNA in primary tumors of thyroid cancer using an ultrasensitive mutation assay
Source: BMC Cancer. 2020 May 1;20:368. doi: 10.1186/s12885-020-06862-w (PMC7195771; doi:10.1186/s12885-020-06862-w)
Supplement: Supplementary file 2 — Additional file 2: Table S2. Improvements of current mRNA-based mutation assay in comparison to the original assay of Extendable blocking probe - reverse transcription (ExBP-RT). [file 12885_2020_6862_MOESM2_ESM.pdf]

Table S2. Improvements of current mRNA-based mutation assay in comparison to the original assay of Extendable blocking probe – reverse transcription (ExBP-RT).

	**Current mRNA-based mutation assay (1)**	**Original ExBP-RT (2)**	**Advantages of (1) compared to (2)**
**Reverse enzyme**	Warm-start reverse transcriptase that enables the manipulation of closed tube reverse transcription reactions	Manual hot-start reverse transcriptase via opening the tube on the heating block	More convenient for assay handling in routine clinical settings
**Design of the mutation-specific primer**	A new design of the mutation-specific primer with a shorter 5'-tail sequence (10 nucleotides in length)	The mutation-specific primer with a long 5'-tail sequence (24 nucleotides in length)	For higher sensitivity and improved specificity
**Sensitivity (%)**	0.005	0.017	More sensitive in detection of *BRAF*^{V600E} mRNA
